# Methyl 2-(thio­phene-2-carboxamido)­benzoate

**DOI:** 10.1107/S160053681202082X

**Published:** 2012-05-16

**Authors:** Durga Prasad Singh, Seema Pratap, Ray J. Butcher, Sushil K. Gupta

**Affiliations:** a Department of Chemistry, M.M.V., Banaras Hindu University, Varanasi 221 005, India; b Department of Chemistry, Howard University, 525 College Street NW, Washington, DC 20059, USA; c School of Studies in Chemistry, Jiwaji University, Gwalior, India

## Abstract

The title compound, C_13_H_11_NO_3_S, was synthesized from methyl anthranilate, triethyl­amine and 2-thio­phenoyl chloride in benzene. The mol­ecular conformation is stabilized by an intra­molecular N—H⋯O hydrogen bond. The dihedral angle between the rings is 2.74 (12)°. In the crystal, C—H⋯O inter­actions link neighbouring mol­ecules into a three-dimensional network.

## Related literature

For the synthesis, see: Sladowska *et al.* (1980 ▶).
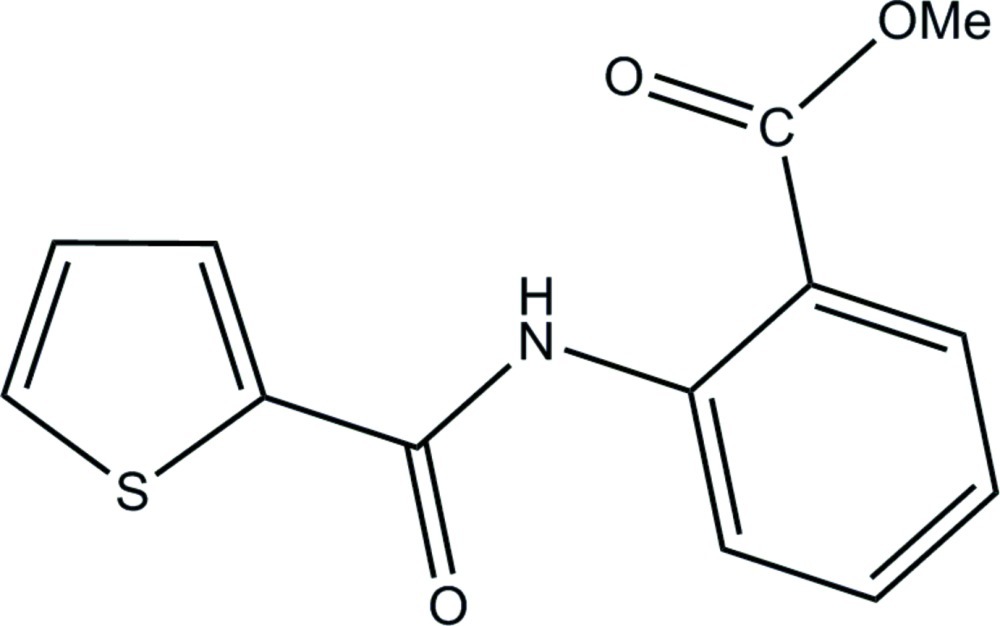


## Experimental

### 

#### Crystal data

C_13_H_11_NO_3_S
*M* *_r_* = 261.29
Orthorhombic, 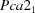

*a* = 19.2845 (4) Å
*b* = 3.86753 (8) Å
*c* = 15.6430 (3) Å
*V* = 1166.71 (4) Å^3^
*Z* = 4
Cu *K*α radiation
μ = 2.48 mm^−1^
*T* = 123 K
0.45 × 0.18 × 0.04 mm

#### Data collection

Agilent Xcalibur Ruby Gemini diffractometer
Absorption correction: analytical (*CrysAlis PRO*; Agilent, 2012 ▶) *T*_min_ = 0.573, *T*_max_ = 0.908
2379 measured reflections
1422 independent reflections
1381 reflections with *I* > 2σ(*I*)
*R*_int_ = 0.033

#### Refinement

*R*[*F* ^2^ > 2σ(*F* ^2^)] = 0.033
*wR*(*F* ^2^) = 0.090
*S* = 1.04
1422 reflections
168 parameters
2 restraints
H atoms treated by a mixture of independent and constrained refinement
Δρ_max_ = 0.21 e Å^−3^
Δρ_min_ = −0.27 e Å^−3^
Absolute structure: Flack (1983 ▶), 206 Friedel pairs
Flack parameter: −0.02 (2)

### 

Data collection: *CrysAlis PRO* (Agilent, 2012 ▶); cell refinement: *CrysAlis PRO*; data reduction: *CrysAlis PRO*; program(s) used to solve structure: *SHELXS97* (Sheldrick, 2008 ▶); program(s) used to refine structure: *SHELXL97* (Sheldrick, 2008 ▶); molecular graphics: *SHELXTL* (Sheldrick, 2008 ▶); software used to prepare material for publication: *SHELXTL*.

## Supplementary Material

Crystal structure: contains datablock(s) I, global. DOI: 10.1107/S160053681202082X/bt5852sup1.cif

Structure factors: contains datablock(s) I. DOI: 10.1107/S160053681202082X/bt5852Isup2.hkl

Supplementary material file. DOI: 10.1107/S160053681202082X/bt5852Isup3.cml

Additional supplementary materials:  crystallographic information; 3D view; checkCIF report

## Figures and Tables

**Table 1:** Hydrogen-bond geometry (Å, °)

*D*—H⋯*A*	*D*—H	H⋯*A*	*D*⋯*A*	*D*—H⋯*A*
N1—H1*B*⋯O2	0.85 (2)	1.99 (3)	2.665 (3)	135 (4)
C9—H9*A*⋯O2^i^	0.95	2.43	3.380 (3)	174
C13—H13*A*⋯O1^ii^	0.98	2.52	3.433 (4)	154

Symmetry codes: (i) 
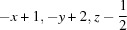
; (ii) 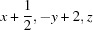.

**Fig. 1.**
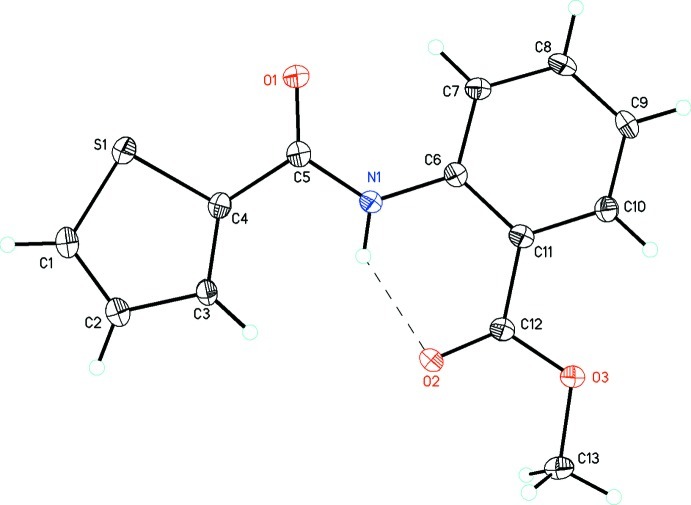
The molecular structure of compound(I) showing the atom labelling scheme. Displacement ellipsoids are drawn at the 30% probability level. The intramolecular hydrogen bond is shown as dashed lines.

**Fig. 2.**
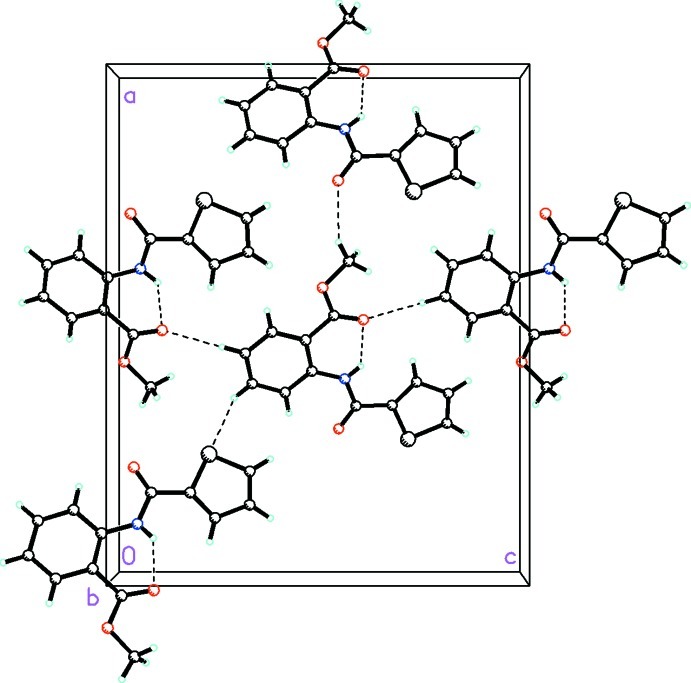
Part of the crystal structure of (I) showing intramolecular and intermolecular interactions as dashed lines.

## supplementary crystallographic information

### Comment

As part of our studies of substituent effects on the structures of amides, we report here the crystal structure of the amide, methyl 2-(thiophene-2-carboxamido)benzoate. The structure of the title compound is shown in Fig. 1. The conformation of the molecule with respect to the carbonyl and anthranilate part is nearly planar as reflected by torsion angles C4—C5—N1—C6, C6—N1—C5—O1 and C3—C4—C5—O1 of 179.6 (2), -0.8 (5) and 178.3 (3) Å respectively. The 2-thiophenoyl and anthranilate groups are *trans* to each other across the C5—N1 bond. Bonds C5—O1 and C12—O2 show typical double bond character with bond lengths of 1.226 (3) and 1.211 (4) respectively, while N1—C5, N1—C6 and C12—O3 show partial double bond character with bond lengths of 1.365 (4), 1.401 (4), and 1.329 (3) Å respectively. All bond length and bond angles confirm the *sp*^2^ hybridization for all C and N atoms except C_13_, indicating that the whole molecule is planar.

### Experimental

The title compound was synthesized using the literature procedure (Sladowska *et al.*, 1980). To a solution of methyl anthranilate (10 mmol) and triethyl amine(10mmol) in benzene(30 ml) was added 2-thiophenoyl chloride (10 mmol) in benzene(10 ml) with stirring at room temperature. After stirring for three hour, the reaction mixture was washed successively with water, dilute HCl and aqueous Na_2_CO_3_ and the organic layer was dried over dry Na_2_SO_4_. After removal of the solvent, the residue was recrystallized from ethanol. Colorless, needles type crystal suitable for X-ray diffraction were obtained after few days. Yield 78%.

### Refinement

H atoms were placed in calculated positions with C—H = 0.95–0.98 Å with isotropic displacement parameters fixed to *U*_iso_(H) = 1.2 *U*_eq_(C). The H attached to N was isotropically refined but with the N—H distance restrained to 0.88 Å.

### Crystal data

**Table:**

C_13_H_11_NO_3_S	*F*(000) = 544
*M**_r_* = 261.29	*D*_x_ = 1.488 Mg m^−^^3^
Orthorhombic, *P**c**a*2_1_	Cu *K*α radiation, λ = 1.54184 Å
Hall symbol: P 2c -2ac	Cell parameters from 1440 reflections
*a* = 19.2845 (4) Å	θ = 2.8–75.1°
*b* = 3.86753 (8) Å	µ = 2.48 mm^−^^1^
*c* = 15.6430 (3) Å	*T* = 123 K
*V* = 1166.71 (4) Å^3^	Needle, colorless
*Z* = 4	0.45 × 0.18 × 0.04 mm

### Data collection

**Table:**

Agilent Xcalibur Ruby Gemini diffractometer	1422 independent reflections
Radiation source: Enhance (Cu) X-ray Source	1381 reflections with *I* > 2σ(*I*)
Graphite monochromator	*R*_int_ = 0.033
Detector resolution: 10.5081 pixels mm^-1^	θ_max_ = 75.2°, θ_min_ = 4.6°
ω scans	*h* = −15→23
Absorption correction: analytical (*CrysAlis PRO*; Agilent, 2012)	*k* = −4→4
*T*_min_ = 0.573, *T*_max_ = 0.908	*l* = −6→19
2379 measured reflections	

### Refinement

**Table:**

Refinement on *F*^2^	Secondary atom site location: difference Fourier map
Least-squares matrix: full	Hydrogen site location: inferred from neighbouring sites
*R*[*F*^2^ > 2σ(*F*^2^)] = 0.033	H atoms treated by a mixture of independent and constrained refinement
*wR*(*F*^2^) = 0.090	*w* = 1/[σ^2^(*F*_o_^2^) + (0.0597*P*)^2^ + 0.0898*P*] where *P* = (*F*_o_^2^ + 2*F*_c_^2^)/3
*S* = 1.04	(Δ/σ)_max_ < 0.001
1422 reflections	Δρ_max_ = 0.21 e Å^−^^3^
168 parameters	Δρ_min_ = −0.27 e Å^−^^3^
2 restraints	Absolute structure: Flack (1983), 206 Friedel pairs
Primary atom site location: structure-invariant direct methods	Flack parameter: −0.02 (2)

### Special details

**Table:**

Geometry. All e.s.d.'s (except the e.s.d. in the dihedral angle between two l.s. planes) are estimated using the full covariance matrix. The cell e.s.d.'s are taken into account individually in the estimation of e.s.d.'s in distances, angles and torsion angles; correlations between e.s.d.'s in cell parameters are only used when they are defined by crystal symmetry. An approximate (isotropic) treatment of cell e.s.d.'s is used for estimating e.s.d.'s involving l.s. planes.
Refinement. Refinement of *F*^2^ against ALL reflections. The weighted *R*-factor *wR* and goodness of fit *S* are based on *F*^2^, conventional *R*-factors *R* are based on *F*, with *F* set to zero for negative *F*^2^. The threshold expression of *F*^2^ > σ(*F*^2^) is used only for calculating *R*-factors(gt) *etc*. and is not relevant to the choice of reflections for refinement. *R*-factors based on *F*^2^ are statistically about twice as large as those based on *F*, and *R*- factors based on ALL data will be even larger.

### Fractional atomic coordinates and isotropic or equivalent isotropic displacement parameters (Å^2^)

**Table:**

	*x*	*y*	*z*	*U*_iso_*/*U*_eq_	
S1	0.25886 (3)	0.20133 (16)	0.72770 (5)	0.03006 (19)	
O1	0.28253 (10)	0.3657 (6)	0.54680 (14)	0.0304 (4)	
O2	0.51080 (11)	0.8922 (6)	0.60945 (13)	0.0339 (5)	
O3	0.57519 (10)	1.1406 (5)	0.50816 (14)	0.0308 (4)	
N1	0.38872 (12)	0.6290 (6)	0.56315 (15)	0.0252 (5)	
H1B	0.4151 (18)	0.698 (9)	0.603 (2)	0.037 (10)*	
C1	0.29286 (16)	0.2186 (7)	0.8287 (2)	0.0307 (6)	
H1A	0.2694	0.1372	0.8781	0.037*	
C2	0.35685 (16)	0.3611 (8)	0.8301 (2)	0.0304 (6)	
H2A	0.3831	0.3919	0.8810	0.037*	
C3	0.38110 (14)	0.4607 (7)	0.74725 (17)	0.0249 (5)	
H3A	0.4251	0.5621	0.7364	0.030*	
C4	0.33202 (13)	0.3900 (7)	0.68499 (18)	0.0251 (5)	
C5	0.33132 (14)	0.4577 (7)	0.59160 (18)	0.0247 (6)	
C6	0.40520 (13)	0.7321 (7)	0.4797 (2)	0.0231 (5)	
C7	0.36094 (14)	0.6720 (7)	0.41074 (19)	0.0262 (6)	
H7A	0.3173	0.5642	0.4201	0.031*	
C8	0.38002 (15)	0.7678 (7)	0.3290 (2)	0.0279 (6)	
H8A	0.3498	0.7185	0.2826	0.034*	
C9	0.44255 (15)	0.9352 (7)	0.31331 (18)	0.0277 (6)	
H9A	0.4550	1.0010	0.2569	0.033*	
C10	0.48604 (14)	1.0039 (7)	0.38091 (19)	0.0261 (6)	
H10A	0.5284	1.1221	0.3708	0.031*	
C11	0.46910 (13)	0.9032 (7)	0.46454 (18)	0.0241 (6)	
C12	0.51909 (14)	0.9743 (7)	0.53544 (18)	0.0253 (5)	
C13	0.62744 (16)	1.2115 (9)	0.5729 (2)	0.0359 (7)	
H13A	0.6645	1.3525	0.5479	0.054*	
H13B	0.6469	0.9929	0.5937	0.054*	
H13C	0.6062	1.3367	0.6206	0.054*	

### Atomic displacement parameters (Å^2^)

**Table:**

	*U*^11^	*U*^22^	*U*^33^	*U*^12^	*U*^13^	*U*^23^
S1	0.0264 (3)	0.0293 (3)	0.0345 (4)	−0.0023 (2)	0.0046 (3)	0.0010 (4)
O1	0.0248 (9)	0.0361 (10)	0.0303 (10)	−0.0051 (8)	−0.0037 (8)	−0.0012 (9)
O2	0.0329 (10)	0.0454 (11)	0.0235 (10)	−0.0104 (9)	−0.0042 (9)	0.0033 (10)
O3	0.0254 (9)	0.0401 (11)	0.0268 (9)	−0.0077 (9)	−0.0033 (9)	0.0017 (9)
N1	0.0234 (10)	0.0295 (11)	0.0227 (11)	−0.0024 (9)	−0.0005 (9)	−0.0013 (9)
C1	0.0342 (14)	0.0284 (14)	0.0294 (15)	0.0026 (11)	0.0072 (12)	0.0021 (12)
C2	0.0341 (14)	0.0299 (13)	0.0272 (14)	0.0021 (12)	0.0037 (12)	0.0013 (12)
C3	0.0281 (12)	0.0242 (11)	0.0224 (12)	−0.0015 (10)	0.0044 (11)	0.0004 (10)
C4	0.0230 (12)	0.0226 (12)	0.0298 (14)	0.0020 (10)	0.0037 (11)	0.0009 (10)
C5	0.0245 (12)	0.0208 (12)	0.0288 (14)	0.0035 (10)	0.0019 (11)	0.0001 (10)
C6	0.0239 (13)	0.0211 (11)	0.0243 (12)	0.0017 (9)	0.0006 (11)	−0.0002 (11)
C7	0.0232 (12)	0.0263 (13)	0.0290 (15)	−0.0009 (10)	−0.0042 (12)	−0.0018 (11)
C8	0.0301 (13)	0.0282 (12)	0.0255 (14)	0.0035 (11)	−0.0094 (12)	−0.0020 (11)
C9	0.0333 (13)	0.0287 (14)	0.0210 (12)	0.0050 (11)	0.0007 (11)	0.0033 (11)
C10	0.0256 (12)	0.0251 (13)	0.0277 (14)	0.0007 (11)	0.0018 (11)	0.0005 (10)
C11	0.0239 (12)	0.0218 (11)	0.0267 (14)	0.0028 (10)	−0.0032 (11)	−0.0021 (11)
C12	0.0240 (12)	0.0260 (12)	0.0258 (13)	0.0006 (10)	−0.0017 (10)	−0.0011 (10)
C13	0.0292 (14)	0.0416 (17)	0.0370 (16)	−0.0082 (12)	−0.0090 (14)	0.0002 (15)

### Geometric parameters (Å, º)

**Table:**

S1—C1	1.711 (3)	C4—C5	1.484 (4)
S1—C4	1.723 (3)	C6—C7	1.395 (4)
O1—C5	1.226 (3)	C6—C11	1.419 (3)
O2—C12	1.211 (4)	C7—C8	1.381 (4)
O3—C12	1.329 (3)	C7—H7A	0.9500
O3—C13	1.454 (4)	C8—C9	1.390 (4)
N1—C5	1.365 (4)	C8—H8A	0.9500
N1—C6	1.401 (4)	C9—C10	1.375 (4)
N1—H1B	0.852 (19)	C9—H9A	0.9500
C1—C2	1.352 (4)	C10—C11	1.404 (4)
C1—H1A	0.9500	C10—H10A	0.9500
C2—C3	1.431 (4)	C11—C12	1.495 (4)
C2—H2A	0.9500	C13—H13A	0.9800
C3—C4	1.385 (4)	C13—H13B	0.9800
C3—H3A	0.9500	C13—H13C	0.9800
			
C1—S1—C4	91.59 (15)	C8—C7—H7A	119.7
C12—O3—C13	115.6 (3)	C6—C7—H7A	119.7
C5—N1—C6	128.7 (2)	C7—C8—C9	121.3 (3)
C5—N1—H1B	113 (3)	C7—C8—H8A	119.4
C6—N1—H1B	117 (3)	C9—C8—H8A	119.4
C2—C1—S1	112.4 (2)	C10—C9—C8	118.9 (3)
C2—C1—H1A	123.8	C10—C9—H9A	120.6
S1—C1—H1A	123.8	C8—C9—H9A	120.6
C1—C2—C3	113.2 (3)	C9—C10—C11	121.4 (2)
C1—C2—H2A	123.4	C9—C10—H10A	119.3
C3—C2—H2A	123.4	C11—C10—H10A	119.3
C4—C3—C2	111.1 (2)	C10—C11—C6	119.2 (2)
C4—C3—H3A	124.4	C10—C11—C12	119.4 (2)
C2—C3—H3A	124.4	C6—C11—C12	121.4 (3)
C3—C4—C5	131.5 (2)	O2—C12—O3	122.8 (3)
C3—C4—S1	111.7 (2)	O2—C12—C11	125.2 (3)
C5—C4—S1	116.7 (2)	O3—C12—C11	112.1 (2)
O1—C5—N1	125.2 (3)	O3—C13—H13A	109.5
O1—C5—C4	121.2 (3)	O3—C13—H13B	109.5
N1—C5—C4	113.5 (2)	H13A—C13—H13B	109.5
C7—C6—N1	122.3 (2)	O3—C13—H13C	109.5
C7—C6—C11	118.6 (3)	H13A—C13—H13C	109.5
N1—C6—C11	119.1 (3)	H13B—C13—H13C	109.5
C8—C7—C6	120.6 (3)		
			
C4—S1—C1—C2	0.0 (2)	C11—C6—C7—C8	2.0 (4)
S1—C1—C2—C3	−0.5 (3)	C6—C7—C8—C9	−1.9 (4)
C1—C2—C3—C4	0.8 (4)	C7—C8—C9—C10	0.3 (4)
C2—C3—C4—C5	177.1 (3)	C8—C9—C10—C11	1.2 (4)
C2—C3—C4—S1	−0.8 (3)	C9—C10—C11—C6	−1.1 (4)
C1—S1—C4—C3	0.4 (2)	C9—C10—C11—C12	178.1 (2)
C1—S1—C4—C5	−177.8 (2)	C7—C6—C11—C10	−0.5 (4)
C6—N1—C5—O1	−0.8 (5)	N1—C6—C11—C10	179.4 (3)
C6—N1—C5—C4	179.6 (2)	C7—C6—C11—C12	−179.7 (2)
C3—C4—C5—O1	178.3 (3)	N1—C6—C11—C12	0.2 (4)
S1—C4—C5—O1	−3.9 (4)	C13—O3—C12—O2	1.5 (4)
C3—C4—C5—N1	−2.1 (4)	C13—O3—C12—C11	−178.4 (2)
S1—C4—C5—N1	175.66 (19)	C10—C11—C12—O2	−178.1 (3)
C5—N1—C6—C7	1.1 (4)	C6—C11—C12—O2	1.0 (4)
C5—N1—C6—C11	−178.8 (3)	C10—C11—C12—O3	1.7 (4)
N1—C6—C7—C8	−177.9 (3)	C6—C11—C12—O3	−179.1 (2)

### Hydrogen-bond geometry (Å, º)

**Table:**

*D*—H···*A*	*D*—H	H···*A*	*D*···*A*	*D*—H···*A*
N1—H1*B*···O2	0.85 (2)	1.99 (3)	2.665 (3)	135 (4)
C9—H9*A*···O2^i^	0.95	2.43	3.380 (3)	174
C13—H13*A*···O1^ii^	0.98	2.52	3.433 (4)	154

Symmetry codes: (i) −*x*+1, −*y*+2, *z*−1/2; (ii) *x*+1/2, −*y*+2, *z*.
